# Reliability and validity of a Dutch version of the Leicester Cough Questionnaire

**DOI:** 10.1186/1745-9974-3-3

**Published:** 2007-02-21

**Authors:** Arnold N Huisman, Mei-Zei Wu, Steven M Uil, Jan Willem K van den Berg

**Affiliations:** 1 Department of Pulmonology, Isala klinieken, Postbus 10500, 8000 GM Zwolle, The Netherlands; 2 University Medical Center Groningen, University of Groningen, Groningen, the Netherlands

## Abstract

**Background:**

Chronic cough is a common condition with a significant impact on quality of life. Currently, no health status measure specific for chronic cough exists in the Netherlands. Thus we developed a Dutch version of the Leicester Cough Questionnaire (LCQ) and tested its scaling and clinical properties.

**Methods:**

The LCQ was adapted for Dutch conditions following a forward-backward translation procedure. All patients referred to our cough clinic between May 2004 and February 2005 completed five questionnaires, the LCQ, the modified Borg score for cough, the Short-Form 36 (SF-36), the Hospital Anxiety and Depression Scale (HADS) and the Global Rating of Change (GRC) upon presentation, after two weeks and after 6 months. Concurrent validation, internal consistency, repeatability and responsiveness were determined.

**Results:**

For the concurrent validation the correlation coefficients (n = 152 patients) between the LCQ and the other outcome measures varied between 0.22 and 0.61. The internal consistency of the LCQ (n = 58) was high for each of the domains with a Crohnbach's alpha coefficient between 0.77 and 0.91. The two week repeatability of the LCQ in patients with no change in cough (n = 48) was high with intraclass correlation coefficients varying between 0.86 and 0.93. Patients who reported an improvement in cough (n = 140) after 6 months demonstrated significant improvement on each of the domains of the LCQ.

**Conclusion:**

The Dutch version of the LCQ is a valid and reliable questionnaire to measure (changes of) health status in patients with chronic cough.

## Background

Chronic cough, defined as cough lasting more than 8 weeks, is a common condition with an estimated prevalence of 20–40%[1,2]. Approximately 10% of the new patients seen in outpatient clinical settings were referred to the pulmonologist because of cough[3].

Chronic cough can be highly disturbing to the patient and its environment, and determining the cause of cough may be difficult. The three most common causes of cough are asthma, gastroesophageal reflux disease and rhinosinusitis. By utilising a systematic protocol for investigation and treatment of cough, it has been reported that in up to 80 to 100% of patients with cough a cause can be identified and patients can be adequately treated[4].

This "anatomic and diagnostic" protocol relies on the most common causes of cough and has been described more than 25 years ago[5]. We introduced a comparable protocol in May 2004 at our hospital, thus starting the first cough clinic in the Netherlands.

Quality of life is an important outcome parameter in Dutch studies on asthma, COPD, lung cancer and lung transplantation [6-9]. Research on quality of life in patients with chronic cough has been performed only recently [10-12]. However, a quality of life questionnaire in Dutch specific for cough did not exist yet.

Therefore, the aim of this study was to develop a Dutch version of the Leicester Cough Questionnaire (LCQ) and to confirm its reliability, validity and responsiveness.

## Methods

### The Leicester Cough Questionnaire (LCQ)

The LCQ is a cough specific quality of life questionnaire with 19 items. It is designed for self-administration and takes less than 5 minutes for completion. The 19 items are divided into 3 domains: physical, psychological and social. A 7-point Likert scale is used to evaluate the answers; a higher score indicates a better health status. The total score is the sum of the scores of the three domains (varying 1 to 7). The LCQ already has been validated in English and has also been used in at least one other language[11,13].

### Patients

All patients with chronic cough referred to our tertiary cough clinic between May 2004 and February 2005 were asked to participate by completion of the questionnaires at the first visit, after 2 weeks and after 6 months. Chronic cough was defined as a cough lasting more than 8 weeks that remained unexplained after assessment by the primary care physician.

### Questionnaires

We used the LCQ, the Short Form36 (SF36), a generic quality of life questionnaire[14], the Hospital Anxiety and Depression Scale (HADS), a questionnaire to detect mild forms of depression and anxiety[15], a modified Borg score for cough scoring the intensity on a scale from 0 (no cough at all) to 10 (maximum cough) and a questionnaire to quantify the degree of change in cough (global rating of change: GRC). The GRC assessment was done to evaluate self-perceived changes in disease control since the first visit. Responses were scored from +7 (a very great deal better) to -7 (a very great deal worse); 0 indicated no change. Scores of -3, -2, +2 and +3 were considered to represent minimal but nevertheless clinically important changes. [16].

### Translation procedure

The translation followed an established forward-backward translation procedure, with independent translations and counter-translation. Independent translations into Dutch of the LCQ (the authors J.B and A.H) were pooled to a common version. A native English speaker fluent in Dutch and with a medical background translated this provisional Dutch version back into English. This back translation was found to be nearly identical to the source document. The Dutch version [see Additional file] was then tested in 4 patients with chronic cough for problems in acceptance and comprehension of the questionnaire content or the phrasing.

### Validation

To validate the LCQ we tested four different aspects of the questionnaire, i.e. the concurrent validity, the internal consistency, the repeatability and the responsiveness. The first two aspects are related to validity, the instrument's ability to measure what it purports to measure[17]. Concurrent validity was tested by comparing the LCQ with other health outcome questionnaires during the first visit. The internal consistency, the degree of homogeneity within a domain, was determined by the degree of correlation between the answers on the questions within a domain.

The repeatability (or test-retest reliability) measures the stability of scores on the LCQ over time. In our patients repeatability was determined by comparing the LCQ scores of the first visit with the LCQ scores after 2 weeks in patients who reported their cough had been unchanged (GRC score = 0).

Responsiveness of a test is the capacity to detect important changes over time[18]. In our study responsiveness was determined by comparing the LCQ scores between the first visit and the LCQ scores after 6 months in patients who told their cough had significantly improved (GRC = 4)

### Statistical analysis

SPSS version 12 was used for data analysis. Data are presented as mean (SE) or ranges. Pearson correlation coefficients between LCQ scores and the scores of the other health outcome were used to determine concurrent validation. Internal consistency was determined by calculating the Cronbach's alpha coefficients for the three domains and the total LCQ. Analysis of the test-retest reliability was done by calculating the Intraclass Correlation Coefficient (ICC) for the three domains and for the total score. Responsiveness was analysed by calculating the 95% confidence interval for the average improvements in the three domain scores and the total score of the LCQ.

## Results

### Patients

The patients' characteristics are shown in table 1. The majority of the patients were female, of middle age.

**Table 1:** Patient characteristics

n		152
Sex m, f (%f)		50, 102 (67%)
Age, years		59 ± 12
Duration of cough, years		5.0
FEV_1 _%predicted		103 ± 21
LCQ	physical	4.4 ± 1.1
	psychological	4.2 ± 1.0
	social	3.8 ± 1.3
	total	12.3 ± 3.0
HADS	anxiety	4.6 ± 3.5
	depression	4.1 ± 3.8
SF-36	general health	57.3 ± 23.2
Borg cough scale		3.6 ± 1.7
Current smoker		8%
Pack-years (min-max)		15 (1–100)

Duration of cough and Pack-years: median value

### Concurrent validity

The correlation coefficients of the concurrent validity, determined in 152 patients, are shown in table 2. Except for two all outcome are statistically significant. Summarised, the correlation coefficients with the Borg Cough Scale, the SF-36 general health and the HAD total score were respectively -0.41, 0.41 and -0.46.

**Table 2:** Concurrent validity

**Validated outcome scales**	**LCQ physical**	**LCQ psychological**	**LCQ social**	**LCQ total**
Borg cough scale	-0.37	-0.38	-0.36	-0.41
HADS anxiety	-0.41	-0.40	-0.33	-0.43
HADS depression	-0.36	-0.36	-0.38	-0.42
HADS total	-0.42	-0.42	-0.39	-0.46
SF-36 general health	0.54	0.28	0.30	0.41
SF-36 vitality	0.61	0.38	0.45	0.55
SF-36 mental	0.39	0.41	0.39	0.45
SF-36 pain	0.46	0.22	0.28	0.36
SF-36 emotional	0.35	0.32	0.16 (NS)	0.30
SF-36 physical	0.49	0.23	0.29	0.37
SF-36 social functioning	0.50	0.38	0.43	0.50
SF-36 physical functioning	0.50	0.24	0.34	0.40
SF-36 health changes	0.11 (NS)	0.22	0.22	0.22

Pearson's correlation coefficients between scores on validated questionnaires (Borg cough scale, SF-36, and HADS) and the domain scores and the total score of the LCQ. All correlation coefficients p < 0.05, unless otherwise described.

### Internal consistency

The Cronbach's alpha coefficients for the physical, psychological, social domains and for the total questionnaire were 0.77, 0.84, 0.83 and 0.93 respectively. A Cronbach's alpha coefficient >0.7 is generally accepted as good.

### Test-retest reliability

The intraclass correlation coefficient (ICC) of the test-retest reliability was 0.93 for the total score. In table 3 the ICCs are shown for the domains and the total score. In addition, the results of the original LCQ are shown.

**Table 3:** Repeatability

**Domain LCQ**	**Intraclass correlation coefficient**		**95%CI**	**p-value**
	**Birring **[11]	**Zwolle**		
Physical	0.93	0.86	0.76–0.92	<0.0001
Psychological	0.90	0.93	0.88–0.96	
Social	0.88	0.93	0.87–0.96	
Total	0.96	0.93	0.87–0.96	

Results of the test-retest reliability analysis. The Intraclass correlation coefficients (ICC) are depicted separately for every domain and for the total questionnaire with the suitable 95% confidence interval (95%CI).

### Responsiveness

The LCQ scores improved significantly after treatment. Results are shown in table 4.

**Table 4:** Responsiveness

**Domain LCQ**	**Improvement score**	**95%CI**
Physical	1.42	1.14–1.71
Psychological	1.77	1.47–2.06
Social	2.10	1.70–2.49
Total	5.28	4.41–6.15

Results of the responsiveness analysis. The average improvements after 6 months in the domain scores and for the total score of the LCQ are depicted with 95% confidence interval

## Discussion

Our results show that the Dutch version of the LCQ [see Additional file 1] is a valid and reliable instrument to measure quality of life in patients with chronic cough. The relationship between the Dutch LCQ and other QOL parameters was moderate. This is expected when the LCQ is compared against generic tools not specific for cough. The LCQ appears to be highly repeatable and responsive to change and therefore can be used to evaluate the results of interventions of cough clinics. The Dutch version allows us to compare our patients and outcomes to other clinics using the LCQ.

Quality of life can also be measured by another cough specific questionnaire, the cough quality-of-life questionnaire (CQLQ)[10]. The CQLQ comprises 28 items and 6 domains; the answers are scored on a 4-point Likert scale. A higher score indicates a worse quality of life due to cough. The LCQ as well as the CQLQ are both designed for self-administration. Both questionnaires have been compared in one study where they showed a good correlation[13]. In this particular study, no details were provided about the translation procedure or the validation of the Turkish versions.

Quality of life is a subjective parameter. Objective measurement of cough using a 24-hour registration of cough sounds has been reported[19]. This method has not been validated yet. Psychological and social consequences of chronic cough seem to matter more for patients than physical consequences[12]. Therefore we consider the LCQ, a health status measure at the moment as the most important parameter to evaluate chronic cough. It perfectly fits to the patient's perception and there is lack of well-validated reliable objective alternative parameters that are commercially available to quantify the burden of chronic cough.

## Conclusion

In conclusion, the Dutch version of the LCQ is a brief, easy to administer questionnaire and appears to be valid, reliable and highly responsive.

## Competing interests

The author(s) declare that they have no competing interests.

## Authors' contributions

AH and MW participated in the design of the study and helped to draft the manuscript. SU participated in the design of the study and performed the statistical analysis. JB conceived of the study, participated in the design and coordination and drafted the manuscript. All authors read and approved of the final manuscript.

## Copyright

Reprints of questionnaire: the Leicester Cough Questionnaire including the Dutch version is protected by copyright. Reprints are available from corresponding author and that of Ref 11.

## Supplementary Material

Additional File 1 The Dutch version of the Leicester Cough Questionnaire. This questionnaire (in Dutch) is the translation of the Leicester Cough Questionnaire. Click here for file
