# Supplementary material for: Reliability and validity of a Dutch version of the Leicester Cough Questionnaire
Source: Cough. 2007 Feb 21;3:3. doi: 10.1186/1745-9974-3-3 (PMC1804278; doi:10.1186/1745-9974-3-3)
Supplement: Additional File 1 — The Dutch version of the Leicester Cough Questionnaire. This questionnaire (in Dutch) is the translation of the Leicester Cough Questionnaire. [file 1745-9974-3-3-S1.doc]

| Naam: |  |
| --- | --- |
| Geboortedatum |  |
| Bezoeknmmer: |  |
| Datum: |  |

| HOEST VRAGENLIJST© Dr JWK van den Berg | | | | | | | | |
| --- | --- | --- | --- | --- | --- | --- | --- | --- |
| Deze vragenlijst is ontworpen om de gevolgen van het hoesten op diverse aspecten van uw leven te meten. Lees iedere vraag aandachtig en OMCIRKEL het antwoord dat de **afgelopen twee weken** het meest op u van toepassing is (slechts één antwoord per vraag). Beantwoord a.u.b. alle vragen, en zo eerlijk mogelijk. | | | | | | | | |
| In de **afgelopen twee weken**… | | altijd | meestal | heel vaak | regelmatig | af en toe | zelden | nooit |
| 1. | had u door het hoesten last van pijn op de borstkas of in de maag? | 1 | 2 | 3 | 4 | 5 | 6 | 7 |
| 2. | had u door het hoesten last van slijmophoesten? | 1 | 2 | 3 | 4 | 5 | 6 | 7 |
| 3. | bent u door het hoesten vermoeid geraakt? | 1 | 2 | 3 | 4 | 5 | 6 | 7 |
|  | |  |  |  |  |  |  |  |
| In de **afgelopen twee weken**… | | nooit | zelden | af en toe | regelmatig | heel vaak | meestal | altijd |
| 4. | had u het idee het hoesten onder controle te hebben? | 1 | 2 | 3 | 4 | 5 | 6 | 7 |
|  | |  |  |  |  |  |  |  |
| In de **afgelopen twee weken**… | | altijd | meestal | heel vaak | regelmatig | af en toe | zelden | nooit |
| 5. | hoe vaak schaamde u zich voor het hoesten? | 1 | 2 | 3 | 4 | 5 | 6 | 7 |
| 6. | werd u ongerust door het hoesten? | 1 | 2 | 3 | 4 | 5 | 6 | 7 |
| 7. | verstoorde het hoesten uw werk of andere dagelijkse bezigheden? | 1 | 2 | 3 | 4 | 5 | 6 | 7 |
| 8. | bemerkte u dat het hoesten het plezier in het leven vergalde? | 1 | 2 | 3 | 4 | 5 | 6 | 7 |
| 9. | moest u hoesten van verflucht of andere prikkelende luchtjes? | 1 | 2 | 3 | 4 | 5 | 6 | 7 |
| 10. | hoe vaak werd uw nachtrust verstoord door het hoesten? | 1 | 2 | 3 | 4 | 5 | 6 | 7 |
| 11. | hoe vaak hoestte u op een dag? | 1 | 2 | 3 | 4 | 5 | 6 | 7 |
| 12. | voelde u zich gefrustreerd door het hoesten | 1 | 2 | 3 | 4 | 5 | 6 | 7 |
| 13.  **Vervolg op volgende pagina** | was u het hoesten zat? | 1 | 2 | 3 | 4 | 5 | 6 | 7 |
| In de **afgelopen twee weken**… | | altijd | meestal | heel vaak | regelmatig | af en toe | zelden | nooit |
| 14. | was u hees door het hoesten? | 1 | 2 | 3 | 4 | 5 | 6 | 7 |
|  | |  |  |  |  |  |  |  |
| In de **afgelopen twee weken**… | | nooit | zelden | af en toe | regelmatig | heel vaak | meestal | altijd |
| 15. | zat u vol energie? | 1 | 2 | 3 | 4 | 5 | 6 | 7 |
|  | |  |  |  |  |  |  |  |
| In de **afgelopen twee weken**… | | altijd | meestal | heel vaak | regelmatig | af en toe | zelden | nooit |
| 16. | was u bezorgd dat uw hoest door een ernstige ziekte veroorzaakt werd? | 1 | 2 | 3 | 4 | 5 | 6 | 7 |
| 17. | was u bezorgd dat anderen dachten dat u wat ernstigs mankeerde, door uw hoesten? | 1 | 2 | 3 | 4 | 5 | 6 | 7 |
| 18. | verstoorde uw hoest uw praten of een telefoongesprek? | 1 | 2 | 3 | 4 | 5 | 6 | 7 |
| 19. | meende u dat het hoesten vervelend is voor uw partner, familie of vrienden? | 1 | 2 | 3 | 4 | 5 | 6 | 7 |

Gebaseerd op de **Leicester Cough Questionnaire** © Birring
